# Using the RETAIN Tabletop Simulator as a Summative Assessment Tool for Neonatal Resuscitation Healthcare Professionals: A Pilot Study

**DOI:** 10.3389/fped.2020.569776

**Published:** 2020-11-05

**Authors:** Simran K. Ghoman, Maria Cutumisu, Georg M. Schmölzer

**Affiliations:** ^1^Neonatal Research Unit, Center for the Studies of Asphyxia and Resuscitation, Royal Alexandra Hospital, Edmonton, AB, Canada; ^2^Department of Pediatrics, Faculty of Medicine and Dentistry, University of Alberta, Edmonton, AB, Canada; ^3^Center for Research in Applied Measurement and Evaluation, Department of Educational Psychology, Faculty of Education, University of Alberta, Edmonton, AB, Canada; ^4^Department of Computing Science, Faculty of Science, University of Alberta, Edmonton, AB, Canada

**Keywords:** neonatal, simulation and games, simulation, education, resuscitation, tabletop simulator

## Abstract

**Background:** Frequent and objective summative assessment of neonatal healthcare providers is important to ensure high-quality care to patients during neonatal resuscitation. Currently, neonatal resuscitation providers are only individually assessed using an at-home online multiple-choice questionnaire. While simulation-based assessment is preferred, resource constraints limit its widespread uptake. An alternative approach to simulation-based summative assessment is needed. Simulation-based serious games may provide a solution.

**Objective:** The aim of this study was to examine if individual performance on the RETAIN (REsuscitation TrAINing for healthcare professionals) tabletop simulator can be used as a summative assessment of neonatal resuscitation providers, regardless of their prior board game experience.

**Method:** Neonatal healthcare providers were recruited from a tertiary perinatal center to complete a (1) demographic pre-survey, (2) neonatal resuscitation scenario using an open-answer written pre-test, (3) neonatal resuscitation scenario using the RETAIN tabletop simulator, and (4) post-survey measuring usage and attitudes toward board games. Multiple linear regression analyses using the Johnson–Neyman technique were conducted in R to probe the moderation effect of *years of board game* on the relationship between *pre-test* and *game performance*.

**Results:** Twenty Neonatal Resuscitation Program-trained healthcare providers (nurses, nurse practitioners, respiratory therapists, and fellows) were recruited for this study (*n* = 19 females). Participants' mean (standard deviation) pre-test score was 8.35 (1.81) out of a total 16 possible points (52%) and a score of 18 (4.41) out of a total of 40 possible points (45%) using RETAIN. Overall board game experience was 22.5 (12.6) years. Finally, years of board game moderated significantly the relation between the pre-test and game performance (*B* = −0.13, SE = 0.05, beta = −0.48, *t* = −2.77, *p* < 0.05; 95% CI [−0.24, −0.03]). Thus, participants' performance on the two tests (written and simulator) was significantly positively associated, but only for those who reported fewer than 21.5 years of board game experience.

**Conclusion:** This study reports the preliminary results of a pilot study, indicating that the RETAIN tabletop simulator could be used as a simulation-based summative assessment, an enjoyable, low-cost alternative to traditional assessment approaches. RETAIN offers a solution to the need for more frequent and continued assessment of neonatal resuscitation providers.

## Introduction

Each year, over 13 million newborns around the world require cardiorespiratory intervention at birth, yet one million of these infants die (1, 2). In a report conducted by the Joint Commission, deficiencies in healthcare professionals' (HCP) competence were identified as the root cause for half of newborn deaths and injuries during delivery (3). To address this quality gap, ongoing training and assessment of HCPs' neonatal resuscitation performance is needed to improve health outcomes (4).

To achieve these two goals, guidelines recommend a biennial Neonatal Resuscitation Program (NRP) provider course (2). The course requires learners to read the NRP textbook, complete four digital neonatal resuscitation simulations, and pass a multiple-choice test (5). During the in-class portion, learners practice their skills and participate in team-based simulations, facilitated by a trained instructor (2). However, this approach is inefficient and ineffective. First, training with the resource-intense NRP course results in short- and long-term skills and knowledge decay of the neonatal resuscitation algorithm (6, 7). Second, individual summative assessment of HCPs occurs only during the at-home online multiple-choice questionnaire, which can be completed using the NRP textbook. A need exists for a more objective and robust summative assessment of neonatal HCPs.

Summative assessments evaluate learners by measuring their individual performance on a particular task at the end of an instructional unit (8). While simulation-based summative assessment demonstrates competence, resource constraints hinder its widespread uptake (9, 10). Recognizing these barriers, alternative methods like simulation-based serious games may be an alternative (10). Serious games incorporate a challenging goal and scoring system to educate players on useful knowledge or skills, thereby creating an interactive and motivating problem-based learning experience (11). Scientifically evaluated serious games may provide a solution to the need for frequent and effective assessment of NRP providers (12). However, serious games present a novel educational tool, and therefore, their applicability and efficacy for learners from a wide range of game usage, habits, and attitudes remains unknown.

This study examined whether individual performance on the RETAIN (REsuscitation TrAINing for healthcare professionals) neonatal resuscitation tabletop simulator can be used for objective summative assessment of HCPs, regardless of participants' prior board game experience.

## Methods

### Participants and Recruitment

Participants were *n* = 20 HCPs (19 females and 1 male; 8 nurses, 4 nurse practitioners, 4 respiratory therapists, and 4 neonatal fellows) who completed NRP recertification within the last 24 months (Table 1).

**Table 1:** Descriptive information of participants.

**Characteristic**	
Self-reported gender	19 female 1 male
Clinical position	8 neonatal nurses 4 neonatal nurse practitioners 4 respiratory therapists 4 neonatal fellows
Months since last NRP course (months)	Median (IQR): 6 (1–10.5) Mean (SD): 6.8 (6.4)
Neonatal clinical care experience (years)	Median (IQR): 10.5 (3–17) Mean (SD): 11.3 (9.1)
Did you enjoy playing this game? (1–5 Likert scale)	Median (IQR): 4 (4–4.25) Mean (SD): 4.1 (0.6)
Overall board game experience (years)	Median (IQR): 22.5 (11–30) Mean (SD): 22.5 (12.6)

Participants were recruited on-service from the Neonatal Intensive Care Unit at the Royal Alexandra Hospital, Edmonton, Canada—a tertiary perinatal center admitting >350 infants with a birth weight of <1,500 g annually. Participants were recruited from the unit by research coordinators based on if they were interested in participating in this research study and if they had time to participate. We aimed to recruit participants across clinical positions, so that the study sample was representative of a typical resuscitation team on our unit (neonatal nurses primarily attend resuscitations and are aided by doctors, nurse practitioners, and respiratory therapists). Participant recruitment for this pilot study was limited due to HCP availability (i.e., unpredictable and busy schedules) and time requirement for the study session (i.e., the tabletop game simulator took approximately 15–20 min to complete, whereas the entire study session took approximately 30–40 min to complete). The study was approved by the Human Research Ethics Board at the University of Alberta (Pro00085274), and written informed consent was obtained from the HCP participants prior to participation.

### The REsuscitation TrAINing Tabletop Simulator for Healthcare Professionals

**Figure 1:**
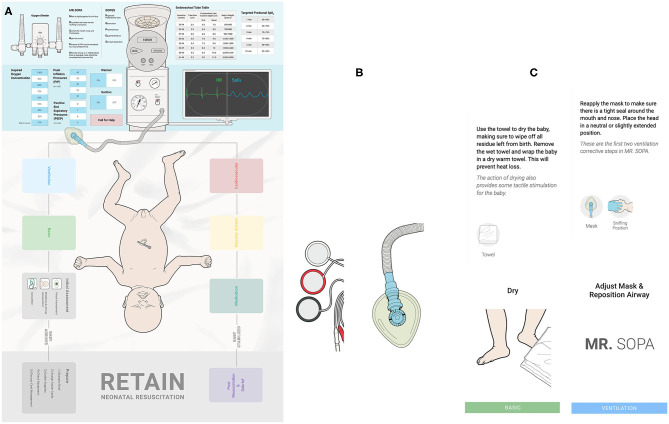
The RETAIN tabletop simulator, including the **(A)** game board, **(B)** equipment pieces, and **(C)** action cards.

The RETAIN tabletop simulator (RETAIN Labs Medical Inc. Edmonton, Canada; https://www.retainlabsmedical.com) was developed for HCPs to practice their knowledge of the neonatal resuscitation algorithm, communication, and teamwork (12–16). The game board consists of an image of a newborn infant, equipment pieces (e.g., radiant warmer), and reference tables (i.e., MR SOPA) (Figure 1A). Players take on the role of an HCP attending deliveries, using 3D equipment and supply pieces, action cards, and adjustable monitors (i.e., time elapsed, or heart rate) to perform interventions (Figures 1B,C). At the end of each scenario, players use debrief cards to reflect on their performance. RETAIN contains over 50 neonatal resuscitation scenarios, based on real cases from the Royal Alexandra Hospital (Edmonton, Canada) delivery room. Further information about RETAIN and gameplay can be found in the literature (12–18).

### Study Design

Each participant completed a questionnaire (e.g., time elapsed since the last NRP recertification) and an open-answer neonatal resuscitation pre-test. The pre-test (Appendix 1) consisted of a neonatal resuscitation scenario of a 24-week premature infant. Participants were instructed to answer the open-ended prompts by explaining the steps they would take to resuscitate and stabilize the infant. After completing the pre-test, participants received instructions on how to play RETAIN, and each participant independently completed one game scenario. The game scenario (Appendix 2) consisted of a neonatal resuscitation scenario of a term infant with fetal bradycardia. More information about the questionnaire is presented in Appendix 3.

### Measures

The pre-test and the game performance were scored using the 7th edition NRP textbook (2).

The *pre-test* measure represents a participant's cumulative score across all actions, interventions, or tasks described by the participant in the written test. The maximum score for each participant, when answering all actions, interventions, and tasks correctly, was 16 points, with a range from 0 to 16. For each action, intervention, or task on the pre-test, participants were assigned one point for a correct answer and were deducted one point for each incorrect answer.

The *game performance* represents a participant's cumulative score across all actions, interventions, or tasks described by the participant in RETAIN. The maximum score for each participant, when answering all actions, interventions, and tasks correctly, was 40 points, with a range from 0 to 40. The game sessions were audio- and video-recorded, and performance was later coded and assessed using the footage by a trained reviewer. Similar to the scoring of the pre-test, for each action, intervention, or task on the game task, participants were assigned one point for a correct answer and were deducted one point for each incorrect answer.

The *years of board game* measure represents the participant's number of years of experience playing board games captured using a constructed-response (i.e., open-text) item: “Please enter your overall board game experience in years.” (as shown in Table 1).

The *enjoyment* measure represents the participants' self-reported enjoyment of playing RETAIN as an answer to the question “Did you enjoy playing this game?” on a 1–5 Likert scale (ranging from 1 = strongly disagree to 5 = strongly agree), as shown in Table 1. Data are presented as mean and standard deviation (SD) or as median and interquartile range (IQR).

### Statistical Analysis

All analyses were performed using the R open statistical computing environment version 3.6.3 (19). We conducted multiple linear regression analyses to test a potential interaction effect of *pre-test* and *years of board game* predicting *game performance*. We employed the *jtools* R package (20) using the Johnson–Neyman technique to determine the regions of significance for the interaction effect, as all our variables were continuous. As different media were used for the assessment (i.e., a traditional paper-and-pencil to measure the *pre-test* and RETAIN to measure *game performance*), we conducted a moderation analysis to partial out the influence of participants' prior gaming experience on the relation between *pre-test* and *game performance*. We mean-centered the independent variable and the moderator before conducting the analyses, for ease of result interpretation.

## Results

The mean (SD) *pre-test* score was 8.35 (1.81) out of 16 (52%), the mean *game performance* score was 18 (4.41) out of 40 (45%), the mean *years of board game* was 22.5 (12.64) out of 50 (45%), and the mean *enjoyment* was 4.1 (0.6) out of 5 (82%). All variables (except for enjoyment) were normally distributed, as shown by non-significant Shapiro–Wilk normality tests. Although the *pre-test* and *game performance* scores were moderately associated, this correlation did not reach significance, possibly due to the dataset being quite small (*r* = 0.39, *p* = 0.08, *n* = 20). Similar results were observed for the relationships between *years of board game* with the *pre-test* (*r* = 0.06, *p* = 0.81, *n* = 20) and the *game performance* (*r* = 0.45, *p* = 0.06, *n* = 20).

**Figure 2:**
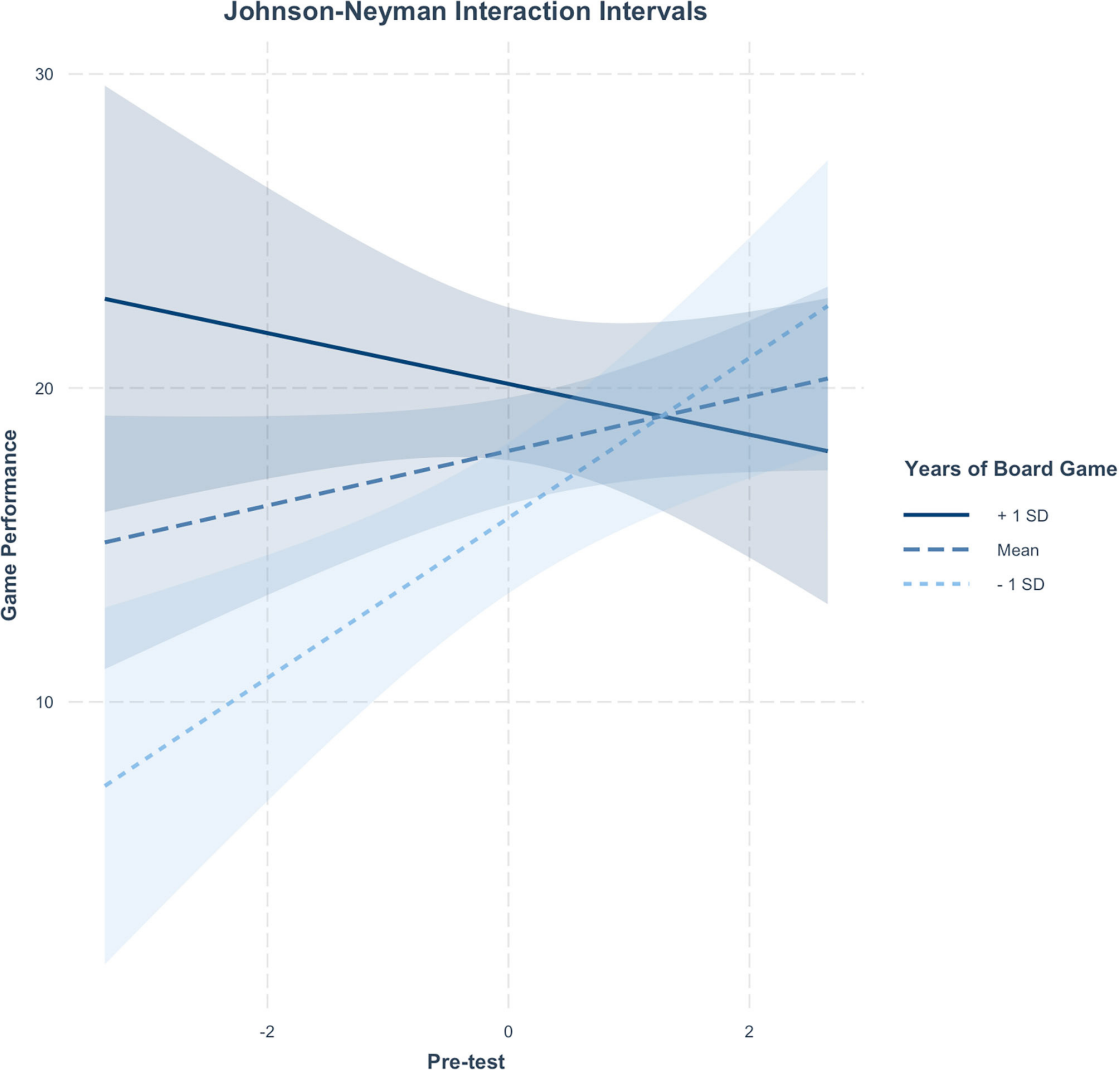
Probing interactions with the Johnson–Neyman (J-N) technique for continuous variables. The range of observed values of *years of board game* is [−19.50, 27.50] and it is represented by a solid black line.

The moderation analysis (21) showed that the model consisting of the independent variable *pre-test*, the moderator *years of board game*, and the interaction between these two variables predicting the dependent variable *game performance* is significant [*F*_(3, 14)_ = 6.62, *p* < 0.01, *R*^2^ = 0.59, adjusted *R*^2^ = 0.50], explaining 50% variance in game performance. *Years of board game* moderated significantly the relation between the *pre-test* and *game performance* (*B* = −0.13, SE = 0.05, beta = −0.48, *t* = −2.77, *p* < 0.05; 95% CI [−0.24, −0.03]), as shown in Figure 2. There was a main effect of *years of board game* predicting *game performance* (*B* = 0.17, SE = 0.06, beta = 0.46, *t* = 2.66, *p* < 0.05; 95% CI [0.03, 0.31]). The interaction means that, every time a person's board game experience increases by 1 year, the adjusted effect of the *pre-test* on *game performance* decreases by 0.13. The results indicate that the better the HCPs performed on the pre-test, the better they performed on RETAIN, but only when they reported lower experience with board games (i.e., fewer than 21.5 years).

**Figure 3:**
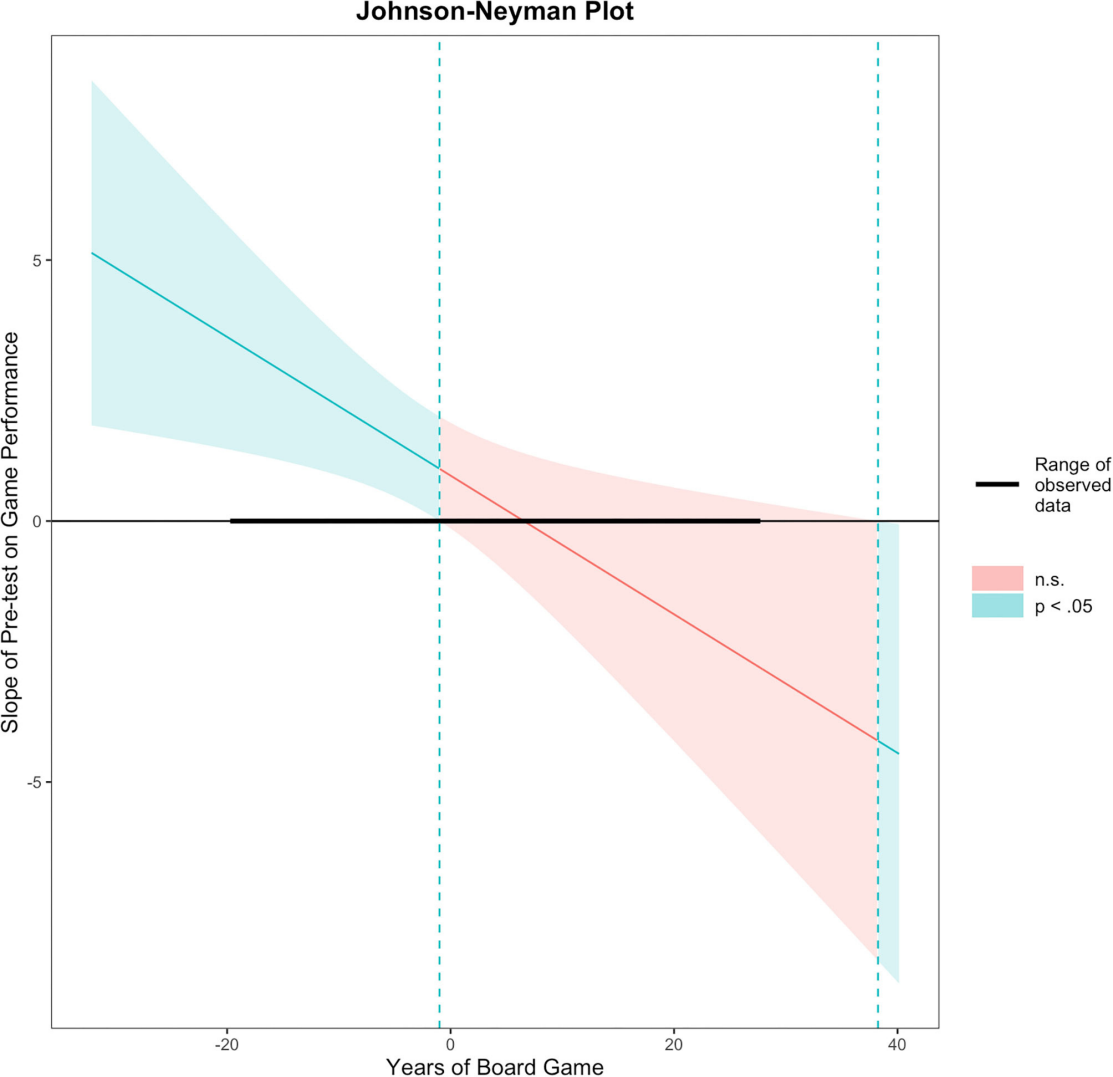
Johnson–Neyman interaction effect of the linear regression model. Participants' performances were significantly associated only when they reported lower *years of board game* experience.

The *y*-axis represents the conditional slope of the pre-test, while the *x*-axis represents units of standard deviation of the *years of board game* moderator. This figure shows where the conditional slope differs significantly from zero: the light blue area on the left of the vertical dotted line between the 95% confidence bands represents the region of significance where the effect of the *pre-test* on the *game performance* is significantly moderated by *years of board game* (i.e., this effect only exists when *years of board game* is lower than or equal to −0.99 represented by the vertical dotted line). The slope of the *pre-test* was significant (*p* < 0.05) when *years of board game* was outside the interval [−0.99, 27.50]. As Figure 3 shows, the maximum observed value was 27.50 for the *years of board game* centered variable (i.e., corresponding to 50 for the non-centered variable). Thus, the slope was significant just below the mean (which in this case coincided with the median) of 22.5 years of board game experience reported (i.e., precisely below 21.5074 years of board game experience). The *x*-axis represents the centered variable *Pre-test* and the *y*-axis represents the *game performance* on RETAIN.

The moderator value defining Johnson–Neyman significance regions was −0.9926. Thus, the better the participants performed on the written test (i.e., the *pre-test*), the better they performed on RETAIN, but only when they reported fewer than 21.5 years of board game experience. There was no significant relationship between the performance on the written test and that on RETAIN when participants reported more than 21.5 years of board game experience. When *years of board game* is 9.86 (i.e., one standard deviation or −12.64 below the mean of the sample, given that this variable is mean-centered), the estimated slope of *pre-test* is 2.55, SE = 0.71, *t* = 3.6, *p* < 0.001 (conditional intercept estimate = 16.52, SE = 1.13, *t* = 14.63, *p* < 0.001). For the average of *years of board game*, the slope is marginally significant (*p* = 0.09). In sum, the results indicate that participants' performances were positively associated only when they reported fewer than 21.5 years and the association strengthened with fewer reported years (for those who reported below 9.86 years of board game experience, the slope was significant at *p* < 0.001).

## Discussion

Lifelong assessment of HCPs' competence is necessary to ensure that the quality of healthcare delivered is congruent with expected standards and to improve health outcomes (4). Neonatal resuscitation demands frequent training by HCPs to combat the knowledge and skills decay observed immediately after completing the NRP provider course (6). Frequent and objective individual summative assessment of neonatal HCPs addresses this issue. However, to facilitate uptake, a feasible and accessible tool is needed. Serious games may offer an attractive alternative.

Summative assessment evaluates learning through performance demonstrated at the end of an instructional unit. The assessment indicates to the learner their relative position in comparison to others or their absolute position in comparison to an expected standard (8, 22). However, traditional high-stakes summative assessment methods may have detrimental effects. First, if the assessment method is not aligned with the content tested (e.g., a multiple-choice exam testing skill performance), HCPs could focus on getting higher exam results and instructors may educate for exams rather than for clinical preparedness (8, 23). Second, feedback during traditional summative assessment is often limited and learners may not have an opportunity to use it due to summative assessments being administered at the end of a learning unit (8, 23). Poorly designed feedback in some summative assessment methods detracts from motivation and depth of learning (8). Methods that focus on clinical practice assessment such as simulations are, therefore, preferred (10, 24).

Simulation-based clinical scenarios are the best method to demonstrate and measure HCPs' learning (10). While simulation is frequently used in training, it remains an underreported approach to assess HCPs' ongoing competency, such as during recertification (10). Barriers to simulation use include the resource-heavy requirements of a high-fidelity manikin, simulation lab, and trained instructor. Also, simulation can be logistically challenging, as multiple learners and instructors must take time off from work to attend the scheduled sessions. Serious games like RETAIN may address these barriers (9, 10).

In this study, we explored the relation between neonatal HCPs' performance on a traditional summative assessment method (open-answer pre-test) and their individual performance on the RETAIN tabletop simulator. We found that HCPs' performance on the pre-test was moderately associated with the performance on RETAIN, but this association did not reach significance, likely because of the small size of our sample. Another alternative explanation of this result is that the pre-test was considerably less difficult than the game scenario. Future research will replicate this study using scenarios of equivalent difficulty to entangle these factors. Similarly, HCPs' game performance was marginally related to years of board game experience, with better game performance with more years of board game experience. We explored whether the game environment played an important role in participants' performance. Specifically, we investigated whether reporting more years of board game experience helped those who achieved a lower performance on a traditional open-answer neonatal resuscitation scenario pre-test perform better on RETAIN than those with fewer years of board game experience.

The moderation analyses explored whether HCPs who performed better on the pre-test maintained their performance on RETAIN, regardless of their years of board game experience. As the moderation effect was significant, results showed that the game can be used as a summative assessment in training and education, as it is positively correlated with other measures of neonatal resuscitation performance, such as the open-answer pre-test, when HCPs reported fewer than 21.5 years of board game experience. This implies that board games do not seem to be an impediment in assessing neonatal resuscitation, as those who performed well on the written test also performed well on RETAIN, especially if they reported fewer years of board game experience. More research is needed to explore the effectiveness of the board game in being used as a summative assessment in many situations (low-stakes and high-stakes) and to further investigate the relation between performance assessed with two media (written test and RETAIN) when participants report more than 21.5 years of experience with board games. As we currently did not find any relation between the two performances for those experienced in playing board games, this could imply that the medium may not be important for those participants. Meanwhile, providing an inviting and familiar assessment environment such as a board game may reveal hidden participant performance that would have otherwise be overlooked by traditional assessments. Overall, players reported enjoying the game. This suggests that RETAIN could be used as a more attractive alternative for assessment. Finally, for participants who are not experienced in playing board games, both assessment methods seem to work equally well.

Cutumisu et al. previously reported that training with RETAIN improves knowledge retention of the neonatal resuscitation algorithm by 12% (13). However, as no help was given to participants during the game scenario, RETAIN can be regarded as a true summative assessment rather than formative assessment. Further, while players could see all the prompts on the action cards while playing, similar conduits are present during traditional simulation as well (such as the NRP algorithm flowchart posted by the bedside).

A limitation of the study was that the pre-test scenario was easier than the game scenario (intermediate versus difficult, respectively). It may have been more effective to compare performance on the two methods using comparable scenarios out of the same number of possible points. Also, the game requires a minimum of two people (player and facilitator) to play, while an open-answer test requires only the learner. However, traditional simulation-based training and assessment require the same, if not more, personnel as the game.

As previously mentioned, only 20 HCPs were recruited, and the limited number of data points precluded deeper analysis, such as between subgroups analyses. It is possible that, with more participants, the marginal correlation between participants' performance on the pre-test and game could become significant. However, this is only the first study to examine if the RETAIN tabletop simulator, which was primarily designed to train HCPs in neonatal resuscitation, can also be used as a summative assessment. The results from this study indicate RETAIN as a promising tool to assess how well learners are prepared for the NRP provider course, as a final assessment at the end of the recertification course or for continuous assessment of HCPs' competence. This study is one step in the ongoing process of validating RETAIN as a comprehensive and effective tool for training and assessing neonatal resuscitation HCPs.

Some of the main benefits of serious games are their characteristics of scalable, accessible, and low-cost distribution to HCPs. Serious games can be played without heavy ongoing resources, advance planning, nor significant time commitment (12). The RETAIN tabletop simulator not only provides an accessible supplement to traditional simulation-based education training but may also be used as a summative assessment tool and, therefore, offers an overall resource-efficient investment. This tabletop simulator also provides ample opportunities to support and assess collaboration among neonatal resuscitation team members, a future research direction that we will pursue.

## Conclusions

The RETAIN tabletop simulator can be used as a summative assessment tool as an enjoyable, clinically relevant, and low-cost alternative to address the need for continual assessment of HCPs' neonatal resuscitation performance. Years of board game experience moderated the marginal relation between performance on the pre-test and on the game, showing that RETAIN can be used as a replacement for more traditional neonatal resuscitation assessments. Specifically, participants' performance on RETAIN was predicted by their performance on the pre-test, but only for those who reported fewer than 21.5 years of board game experience. However, for all participants, RETAIN may provide an enjoyable and resource-efficient alternative for frequent summative assessment of HCPs' neonatal resuscitation competence. The results of this study constitute a first step in exploring whether learning and assessment aspects of neonatal resuscitation knowledge could be transformed using game-based simulations, such as tabletop games.

## Data Availability Statement

All datasets generated for this study are included in the article/Supplementary Material.

## Ethics Statement

The studies involving human participants were reviewed and approved by The study was approved by the Human Research Ethics Board at the University of Alberta (Pro00085274), and written informed consent was obtained from the HCP participants prior to participation. The patients/participants provided their written informed consent to participate in this study.

## Author Contributions

GS, SG, and MC: conception, drafting of the manuscript, critical revision of the manuscript, and final approval of the manuscript. All authors contributed to the article and approved the submitted version.

## Conflict of Interest

GS has registered the RETAIN board game (Tech ID 2017083) and the RETAIN video game under Canadian copyright (Tech ID – 2017086). GS is the owner of RETAIN Labs Medical Inc., Edmonton, Canada (https://www.retainlabsmedical.com/index.html), which is distributing the game. The remaining authors declare that the research was conducted in the absence of any commercial or financial relationships that could be construed as a potential conflict of interest.

## Abbreviations

HCP: Healthcare professional
IQR: Interquartile range
NRP: Neonatal Resuscitation Program
RETAIN: REsuscitation TrAINing for healthcare professionals
SD: Standard deviation.
