# Supplementary material for: Using the RETAIN Tabletop Simulator as a Summative Assessment Tool for Neonatal Resuscitation Healthcare Professionals: A Pilot Study
Source: Front Pediatr. 2020 Nov 5;8:569776. doi: 10.3389/fped.2020.569776 (PMC7674399; doi:10.3389/fped.2020.569776)
Supplement: Supplementary file 1 [file Data_Sheet_1.pdf]

Appendices

Appendix 1: Open-answer test scenario with answer key

Scenario: Your task is to identify the steps needed to react in the following situation: a newborn baby with fetal distress is brought to you.

Medical History: 26-year-old woman G1P0 with no prenatal care currently at 24 weeks pregnant. The mother received 2 doses of Betamethasone and $MgSO_4$ prior caesarean section due to fetal heart abnormalities. The baby is delivered and receives 60sec of delayed cord clamping. The baby is transferred to the Resuscitation table.

What are the next steps?

1. Hat
2. Wrap
3. Stimulate
4. Visually assess
5. Assess breathing
6. Assess heart rate

The clinical team performs initial assessment: Heart rate (HR) 70/min, blue skin color, and apnea
What are the next steps?

1. Attach SpO_2
2. Attach ECG
3. Attach temp probe
4. Suction
5. Reposition head

After reassessment: Heart rate (HR) 50/min, blue skin color, and apnea
What are the next steps?

1. PPV for 60 seconds

After reassessment: HR>100/min, apnea
What are the next steps?

1. MR SOPA
2. PPV for 60 seconds

After reassessment: HR>120/min, spontaneous breathing with increased work of breathing and grunting
What are the next steps?

1. Start CPAP
2. Admit to NICU

No further actions are required thereafter.

Appendix 2: RETAIN board game scenario with answer key

Scenario: You are called to attend a birth due to fetal bradycardia. How would you prepare for the resuscitation of the baby? As you work, say your thoughts and actions aloud so I will know what you are thinking and doing.

Medical History: You are called to attend a birth due to fetal bradycardia for the last 3 minutes. One baby is expected. The fluid is clear.

1. Pre-brief
2. Assign roles
3. Call for assistance
4. Put on protective equipment
5. Gather supplies
6. Check equipment
7. Set ventilation device

The baby has been born.

1. Visually assess
2. Tactile stimulation
3. Dry
4. Visually assess

The clinical team performs initial assessment: The baby has no tone and is not crying.

1. Cord management
2. Maintain temperature
3. Suction
4. Assess breathing
5. Attach ECG and measure heart rate

After reassessment: Heart rate (HR) 40/min and the baby is not breathing.

1. Initiate PPV
2. Attach pulse oximetry sensor and measure oxygen saturation
3. Attach temperature probe
4. Reassess

After reassessment: Heart rate (HR) is still 40/min, the baby is still apneic.

1. MR. SOPA
2. Continue PPV
3. Reassessment
4. MR. SOPA
5. Continue PPV
6. Reassessment
7. MR. SOPA
8. Reassess

After reassessment: HR is 40/min, not increasing, and no chest movement is observed.

1. MR. SOPA

2. Intubation preparation
3. Confirm tube placement

After reassessment: Color did not change on the CO_2 detector, HR is still 40 bpm.

1. Reintubate
2. Confirm tube placement

After reassessment: Color did change on the CO_2 detector, HR is 40 bpm and not increasing.

1. Call for assistance
2. Oxygen blender to 100%
3. Give chest compressions

After reassessment: After 60 seconds of compressions, HR increases to 70 bpm, but no spontaneous respirations.

1. Stop chest compressions
2. Continue PPV

After reassessment: At 4 minutes after birth, HR is >100 bpm, saturation is 78%, but no spontaneous respirations.

1. Continue PPV

After reassessment: At 5 minutes after birth, HR is >100 bpm, saturation is 90%, and beginning to have some spontaneous respirations.

1. Admit to NICU and update parents

Appendix 3: Common actions, interventions, or task shared between the open-answer test and RETAIN game scenario.

	Open-answer test	RETAIN game scenario
1	Wrap	Maintain temperature
2	Stimulate	Stimulate
3	Assess breathing	Assess breathing
4	Assess heart rate	Measure heart rate
5	Attach SpO_2	Attach pulse oximetry sensor
6	Attach temperature probe	Attach temperature probe
7	Suction	Suction
8	PPV for 60 seconds	Initiate PPV
9	MR SOPA	MR SOPA
10	PPV for 60 seconds	Continue PPV
